# A Sentence Classification–Based Medical Status Extraction Pipeline for Electronic Health Records: Institutional Case Study

**DOI:** 10.2196/77409

**Published:** 2026-03-26

**Authors:** Chuanming Dong, Boris Delange, Alex Poiron, Mohamed El Azzouzi, Clément François, Guillaume Bouzillé, Marc Cuggia, Sandie Cabon

**Affiliations:** 1 Univ Rennes, CHU Rennes, INSERM, LTSI - UMR 1099, F-35000 Rennes, France, Rennes, France, +33 02 23 23 62 20; 2 Kereval (France), Rennes, France

**Keywords:** sentence classification, information extraction, natural language processing, clinical data warehouse, deep learning, large language model, artificial intelligence

## Abstract

**Background:**

Clinical data warehouses store large volumes of unstructured text containing valuable information about patients’ medical status. Traditional extraction systems based on named entity recognition (NER) identify medical terms but often fail to capture the contextual cues needed for accurate interpretation. Existing approaches to context-aware extraction differ in their reliance on expert annotation, computational power, and lexical resources, leading to uneven feasibility across institutions. Combined with heterogeneity in documentation practices and data-sharing restrictions, these limitations hinder the scalability and reuse of trained models. There is thus a need for practical frameworks that can be deployed and adapted locally within medical institutions.

**Objective:**

This study aimed to introduce the Medical Status Extraction Pipeline (MSEP)**,** a methodological framework that extracts patients’ medical status from clinical narratives through sentence classification and supports the local deployment of hybrid extractors, illustrated through an institutional case study.

**Methods:**

MSEP extracts medical status by classifying sentences into predefined categories (presence*,* absence, or unknown) for each targeted condition. The pipeline combines modules for data selection, expert annotation, and model development, with parameters customizable to different settings. It was applied within our institutional environment on 6 conditions: smoking, hypertension, diabetes, heart failure, chronic obstructive pulmonary disease, and family history of cancer, using 12,119 manually annotated sentences from the eHOP Clinical Data Warehouse (Rennes University Hospital). Three types of extractors were compared: fine-tuned CamemBERT, large language model (LLM) prompt, and a rule-based baseline, evaluated through stratified 3-fold cross-validation, measuring precision, recall, specificity, macro *F*-score, balanced accuracy, as well as manual annotation time and model inference speed.

**Results:**

Among the tested approaches, the CamemBERT-based extractor achieved the best overall performance, with macro *F*-scores above 0.94 for 5 of the 6 medical conditions. The study also highlights that when a medical status is very sparsely represented in the training data, rule-based extractors can outperform learned models (average macro *F*-score 0.94 vs 0.73 for family history of cancer). This shows the pragmatic value of choosing the extraction method according to data availability. Manual annotation time per sentence ranged from 1.2 to 2.9 seconds within the pipeline (2.23 to 4.25 seconds for informative sentences), compared with 7.8 to 16.5 seconds for named entity recognition–based systems. In our institutional experiments, the minimum time to complete all pipeline modules, from dataset construction to final extractor refinement, was 8 hours.

**Conclusions:**

In our institutional case study, MSEP enabled rapid construction of datasets and extractors across multiple clinical conditions while reducing the effort required for local development. Its modular and configurable design allowed the adoption of hybrid extraction approaches and adaptation to different resource settings. These features highlight MSEP’s value as a research tool and upstream component that facilitates local deployment of clinical information extraction workflows.

## Introduction

In recent years, a substantial amount of clinical data has been collected from numerous patients and stored in clinical data warehouses (CDWs) [1]. These databases represent a valuable resource for clinical research. In many cases, a significant portion of their content exists as free unstructured texts, such as hospital discharge summaries and emergency reports, which can be difficult to process [2]. Nonetheless, these texts contain valuable information about patients’ medical status that, once extracted, can be leveraged for clinical and epidemiological research [3-5]. Harnessing these unstructured data requires developing natural language processing (NLP) methods specifically adapted to the medical domain. Extensive efforts have been made in this area, as reflected in shared tasks such as the n2c2 (National NLP Clinical Challenges) competitions, which have addressed problems of extracting patients’ family history regarding diseases, smoking, suicide, and drinking [6].

According to a recent review, most NLP approaches for medical information extraction rely on named entity recognition (NER) [2]. At first, rule-based approaches using dictionaries and terminologies to match terms and concepts were proposed [7,8]. However, their reliance on lexical resources and medical expertise for rule construction limits their long-term viability, since rules and vocabularies require regular manual updates to reflect evolving clinical practices. To cope with this, machine learning approaches arose. These methods take advantage of the large amount of medical knowledge in databases and can automate the construction and maintenance of medical information retrieval systems [9]. Its latest branch, deep learning approaches, has improved the ability to capture and use contextual information compared with rule-based and traditional machine learning methods, thanks to their neural network architectures; accordingly, this also makes their performance highly dependent on the availability of sufficient annotated data [10,11].

However, NER-based information extraction approaches present a significant limitation. While NER can effectively extract medical entities (eg, “cancer” and “hypertension”), it cannot capture the contextual information required for a full understanding of the patient’s status. A comprehensive assessment involves interpreting the complete syntactic and semantic structure, including subject (patient or family member), predicate (having or not having), and object (the medical condition), to determine whether a condition is present, absent, or mentioned in a familial context [12-14]. Standard NER captures only the object component, necessitating additional processing by context qualifiers to detect negation, temporality, and subject attribution [6,15-17]. Clinical narratives further amplify these challenges, as they are typically unstructured, context-dependent, and institution-specific, with such cues often expressed at the sentence or discourse level [18,19]. For example, in “Tabagisme pendant 7 ans, reprise récente malgré un sevrage en 2020” (Smoking for 7 y, recent relapse despite cessation in 2020), successful interpretation requires resolving the temporal relation between cessation and relapse to infer that the patient is an active smoker, dependencies that standard NER pipelines, which treat entities and assertions separately, struggle to capture. These linguistic characteristics highlight the need for approaches more capable of sentence-level semantic interpretation.

At the same time, developing context-aware information extraction systems can be resource-intensive. The development and evaluation of NER models require extensive expert annotation to label entity boundaries and associated features within large-scale corpora [20], a process made even more laborious by the sparsity of clinically relevant content [21-23]. With the advancement of deep neural networks, embedding fusion techniques have been adopted to enhance context utilization and improve recognition accuracy in NER tasks [24]. However, their cross-domain applicability is limited by high computational cost and reliance on external resources for comprehensive feature engineering. To overcome the limitations of traditional NER pipelines and handcrafted feature engineering, recent research has shifted toward the use of transformer-based pretrained language models that inherently capture richer contextual representations [25]. Encoder-only language models such as BERT [26] and CamemBERT [27] have demonstrated high performance in domain-specific tasks through fine-tuning on relatively small annotated datasets [28]. Decoder-only large language models (LLMs), such as ChatGPT [29] and Mistral [30], can achieve similar goals via prompt engineering without requiring fine-tuning. However, both approaches still depend on domain-specific expertise for model adaptation [31,32], and decoder-only models introduce substantial computational costs that limit their feasibility for routine institutional deployment [33].

Beyond technical constraints, institutional heterogeneity, including differences in documentation practices, terminologies, and privacy regulations, presents major barriers to developing and sharing information extraction systems across institutions [34-36]. Clinical documentation varies widely across institutions, incorporating nonstandard abbreviations, local terminology, and inconsistent formatting [19,37], which hinders the generalizability and scalability of information extraction models, requiring substantial annotation and model adaptation for each use case [19,38]. The sensitive nature of patient data further restricts access to real-world electronic health records and limits the sharing of pretrained models, due to risks of unintended information leakage [39,40]. Consequently, research teams often need to rebuild extraction systems from scratch, even when addressing similar clinical objectives [16,41]. In light of these limitations, there is a critical need for practical, context-aware information extraction systems that can be developed and reused within institutional boundaries.

To address these limitations, we designed the Medical Status Extraction Pipeline (MSEP) as a methodological framework that supports the local development of hybrid approaches for information extraction. MSEP reframes medical status extraction as a sentence classification task, which directly leverages contextual cues such as negation, temporality, and subject attribution while reducing annotation complexity. In addition, by accommodating rule-based methods, fine-tuned language models, and LLM prompting, MSEP allows institutions to select extraction approaches suited to their data availability and computational resources.

To support its interest, the MSEP has been applied to build extractors of patients’ status for 6 clinically relevant medical conditions [16,42,43] from a French CDW: smoking, hypertension, diabetes, heart failure, chronic obstructive pulmonary disease (COPD), and family history of cancer. This paper first outlines the steps, functions, and requirements of the MSEP pipeline, along with the materials and tools used. Next, we present the evaluation results of extractors built using MSEP and compare them with different medical information retrieval approaches (rule-based methods and prompt-based LLM extraction). Finally, we discuss the operational benefits of MSEP, particularly reduced annotation effort, and its potential use as an upstream research tool within institutional settings.

## Methods

### Overview

The MSEP pipeline classifies sentences in clinical documents as present, absent, or unknown regarding a patient’s medical condition. Available as a Python package (repository link in Multimedia Appendix 1), the framework includes configurable parameters that allow its components to be adapted to local research needs and institutional constraints. In the following sections, we first describe the general design of the pipeline and then detail its implementation within our institutional setting.

### Ethical Considerations

Ethics approval for data reuse was obtained from the Commission Nationale de l'Informatique et des Libertés (agreement number 2206739). This study was conducted on pseudonymized secondary clinical data provided by the Clinical Data Center of Rennes Hospital. In accordance with French regulations and the guidelines of the Commission Nationale de l'Informatique et des Libertés. Patients were informed of the potential use of their data for research purposes through the hospital's standard information notice. All data were pseudonymized by the Clinical Data Center before being transmitted to the research team. No identifying information was accessible at any stage of the analysis. No compensation was applicable or provided to participants.

### Generic Pipeline Design

#### Overview

**Figure 1.**
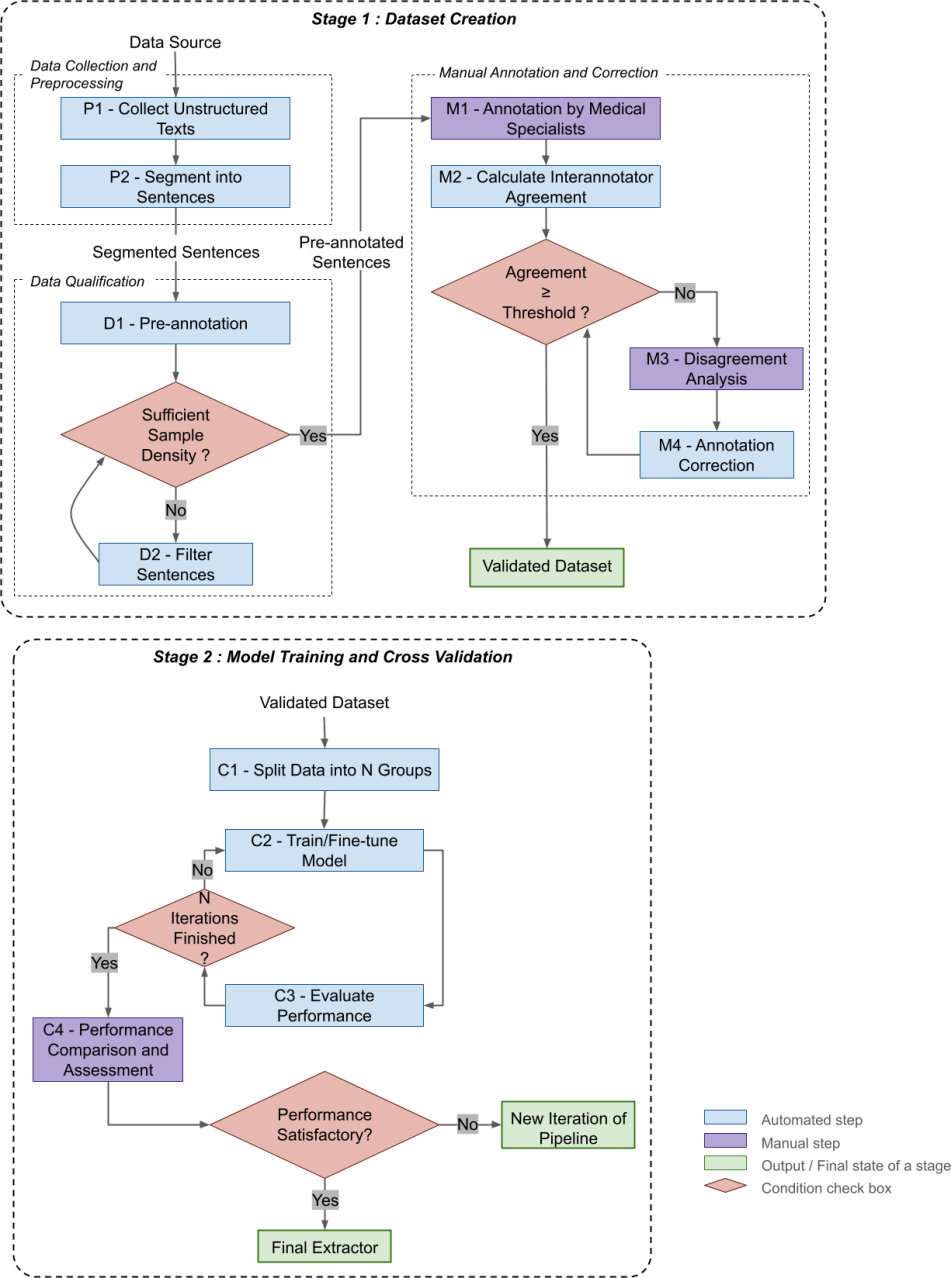
Overview of the proposed iterative medical status extraction system. This pipeline is primarily designed to extract the status of one medical condition at a time; it may repeat multiple times (iterations) to improve the extractors. This diagram mainly shows the process of the first iteration.

The global framework of our method is depicted in Figure 1. The framework consists of two main stages: (1) dataset creation and (2) model training with cross-validation. In order to facilitate the comprehension of the framework, the abbreviations of the steps in the figure are also mentioned in the text.

#### Dataset Creation

##### Overview

The objective of this stage is to construct the datasets required for training and validating medical status extractors. It involves three processes: (1) data collection and preprocessing, (2) data qualification, and (3) manual annotation and correction.

##### Data Collection and Preprocessing

Data collection gathers unstructured texts from CDW (P1), which are preprocessed by segmenting into sentences for classification (P2). The output is a corpus of sentences with relevant metadata that maintains the relationship to their source documents.

##### Data Qualification

The input to the data qualification process is the preprocessed sentence corpus. This phase assesses whether the collected data have appropriate sample density and size for effective model training. Sentences undergo automatic preannotation (D1), preferably using quickly implemented rule-based approaches that match medical status keywords. When preannotating sentences from all documents is impractical due to time and resource constraints, a random subset may be selected. If sample density is low, based on preannotation results, a portion of sentences unlikely to contain medical status information is filtered out (D2). This supervised sampling approach is designed to enhance annotation efficiency and final data quality by optimizing the training sample structure. The output is a preannotated sentence dataset with optimized annotation density and a balanced representation of target medical statuses.

##### Manual Annotation and Correction

The preannotated sentences serve as input for manual annotation, which requires multiple medical specialists to classify them according to annotation guidelines (M1). Annotators should collaboratively annotate a subset of the sentences to calculate interannotator agreement (M2). If the agreement exceeds the predetermined threshold, the annotations form the training and validation datasets. Otherwise, disagreements are analyzed (M3) to improve guidelines, with corrections applied to the annotated data accordingly (M4). The correction approach depends on the type of disagreements, but preferably through manual review. The disagreements analysis-correction process can be repeated until the interannotator agreement exceeds the threshold. The output is a validated annotated dataset ready for model development.

### Model Training and Cross-Validation

The annotated datasets become the input for training or fine-tuning neural network models for medical status extraction. Since the pipeline is designed to extract the status of one medical condition at a time, each corresponding model is trained in a multiclass classification setting, where the possible status labels (eg, present, absent, and unknown) are mutually exclusive. Model stability is assessed through cross-validation by dividing annotated data into several groups (C1), using one group for validation (C3) and the remainder for training (C2) in each session. The number of cross-validation sessions equals the number of groups of data. The data grouping strategy for cross-validation should align with study objectives; for example, testing model stability across different document types requires data partitioning by document type. The extractor’s performance metrics across multiple validation sets are assessed and compared with extractors based on other approaches, if available, to decide whether it has stable and state-of-the-art performance (C4). The flexibility of the pipeline configuration allows for easy benchmarking of different models (such as BERT-based models, rule-based systems, or LLM prompts), facilitating comprehensive performance comparison with minimal additional setup.

If the assessment result is positive, the pipeline ends, and the output is a trained medical status extraction model with satisfactory performance.

### Iterative Improvement Process

If, according to the assessment step (C4), the extractor does not achieve satisfactory performance (eg, a macro *F*-score below 90%), the pipeline allows for iterative refinement. The definition of “satisfactory” performance may vary depending on the objectives and standards of different research projects.

The iterative process involves both quantitative and qualitative improvements. Quantitatively, additional annotated sentences are incrementally introduced to expand the training and validation datasets. The number of added samples per iteration is not fixed and depends on multiple factors, including the time allocated for manual annotation, the number and availability of annotators, and computing resources.

Qualitatively, the pipeline may be refined by improving preannotation rules, updating annotation guidelines based on annotator feedback, or replacing rules with more accurate extractors from previous iterations for preannotation. The first iteration serves as an exploratory phase to identify bottlenecks, such as insufficient data for certain labels, weak vocabulary coverage in preannotation rules, or annotation inconsistencies, allowing for targeted improvements in subsequent iterations.

### Study-Specific Implementation

#### Data Source and Working Corpus

We used the eHOP CDW from Rennes University Hospital, which contains 80 million unstructured and 430 million structured data elements covering over 1.8 million patients [44]. For this study, we extracted unstructured documents from our previous bladder cancer research dataset [3], obtaining 799,470 documents from 5398 patients. This corpus encompasses diverse medical document types including nursing care reports, medical reports, multidisciplinary follow-up & daily notes, imaging reports, prescriptions, and medication orders (Table 1).

**Table 1.** Numbers and ratios of the 9 most frequently found document types (as well as the rest of the 5020 documents in “Other”) in our corpus.

Medical document type	Value, n (%)
Nursing care–procedures	329,359 (41)
Medical reports	187,370 (23)
Nursing care–monitoring and vitals	153,865 (19)
Multidisciplinary follow-up and daily notes	54,187 (7)
Imaging reports	31,430 (4)
Prescriptions and medication orders	13,163 (2)
Paramedical care	13,083 (2)
Administrative and admission	6839 (1)
Results and pathology reports	5225 (1)
Other	5020 (1)

#### Rationale for Extraction Method Design

MSEP is designed as a hybrid framework capable of accommodating different resource-availability scenarios. To assess this flexibility, we evaluated 3 extraction approaches that represent distinct requirements: fine-tuning an encoder-only transformer (high annotation demand and moderate computational cost), rule-based extraction (minimal computation and annotation requirements but substantial reliance on domain expertise), and LLM prompting (low annotation requirements but high computational and expertise demands) [45]. Our experiments focused primarily on encoder-only fine-tuning in order to assess how effectively MSEP can reduce the associated annotation burden in practice, while the rule-based and LLM-prompting approaches served as comparative baselines for low-annotation settings. The rule-based approach is used for preannotation (D1) before fine-tuning, reinforcing the hybrid nature of the MSEP framework.

Regarding the models and tools used in these approaches, CamemBERT was selected for encoder-only fine-tuning strategy because it aligns with the sentence-classification paradigm and has demonstrated strong performance in French NLP tasks [25,46,47]. Furthermore, we opted for the original CamemBERT rather than domain-adapted variants such as CamemBERT-bio [48], as the latter are pretrained on specialized biomedical corpora, which may bias them toward known biomedical tasks and entities [49,50]. Using a general-domain model allows us to evaluate the pipeline’s components without relying on domain-specific pretraining and to examine how it behaves in our institutional corpus when applied to varied clinical conditions. For LLM prompting, we used Mixtral-8×7B-v0.1 [51], which offered the strongest performance that could be efficiently supported by our institutional infrastructure (an NVIDIA A100 40 GB GPU). The rule-based extractors were developed using regular expressions and terminology specific to each targeted medical condition, provided by medical experts.

#### Pipeline Configuration

The pipeline was independently evaluated on 6 medical conditions: smoking, hypertension, diabetes, heart failure, COPD, and family history of cancer. For each condition, we defined 3 possible statuses: “present,” “absent,” and “unknown,” corresponding to confirmed presence, negation, and uncertainty regarding the patient’s condition. For the smoking condition, an additional status, “former,” was included to indicate that the patient previously smoked but has since quit (Table S1 in Multimedia Appendix 2 provides a detailed definition of medical statuses). Multimedia Appendix 2 contains the annotation guidelines, which give more details about the criteria for annotating the medical status.

We used Spacy’s fr_core_news_md model [52] for sentence segmentation (P2) and manually crafted rules based on specialist-provided keywords for preannotation (D1), supplemented by EDS-NLP’s negation and family context qualifiers [53] for smoking and family history of cancer. Two medical specialists performed annotations (M1) using the Prodigy interface [54], with Cohen kappa [55] measuring interannotator agreement (M2), which is defined as:


(1) κ=(Po−Pe)/(1−Pe)

where *Po* is the observed agreement and *Pe* is the expected agreement by chance. A threshold of 0.8 was used to determine acceptable agreement.

For CamemBERT-based extractors, we performed a 3-fold stratified cross-validation. The full annotated dataset was randomly partitioned into 3 equally sized folds (C1), preserving the proportion of annotated categories within each fold. Duplicated (identical) sentences were removed before the partition to prevent data leakage across folds. In each of the 3 cross-validation rounds, two folds were used for training and the remaining fold served as the validation set (C2). We trained the CamemBERT-large model [27] with loss weighting to address class imbalance. The weighted cross-entropy loss with class weighting is defined as:


(2) L=−Σi wi×log p(yi|xi),wi=N/(C×Nyi)

where *p(yi | xi*) is the predicted probability for the true class *yi* of input *xi*, *wi* is the class-specific weight, *N* is the total number of samples, *C* is the number of classes, and *Nyi* is the number of samples in the class of example *i*. Model performance was evaluated (C3) using precision, recall, specificity, *F*-score on individual status, balanced accuracy, and macro *F*-score on all statuses of a medical condition. Balanced accuracy was defined as:


(3) $$\begin{array}{l} Balanced\;accuracy=(1/C)\times \Sigma c[((TPc\;/\;(TPc+FNc))\\ +(TNc\;/\;(TNc+FPc)))/2] \end{array}$$

and macro *F*-score was defined as:


(4) F1macro=(1/C)×Σc F1(c)

where *C* is the number of classes, and *TPc, FNc, TNc,* and *FPc* are respectively the true positives, false negatives, true negatives, and false positives for class *c*. This set of metrics is supposed to cover all necessary aspects of evaluation for an information retrieval system according to [56]. For comparison (C4), we evaluated our rule-based extractors and Mixtral-8x7B-v0.1 LLM prompts on the same validation sets used for the cross-validation (C2+C3). To ensure a quite fair comparison, the Mixtral-8x7B prompts were designed using few-shot examples derived from the annotation guidelines, combining basic extraction instructions with expert-crafted example and counter-example sentences embedding specialist-provided keywords and clinical context narration.

Detailed configuration information for each step in MSEP appears in Multimedia Appendix 1. The MSEP python package (repository link in Multimedia Appendix 1) provides configurable modules to realize each step of the pipeline.

## Results

### Overview

We conducted 2 iterations of the pipeline in our institutional setting to create and refine the extractors, after which most targeted medical conditions reached satisfactory performance (average macro *F*-score>0.94) for our use case. This section presents the results of these iterations, describing implementation paths, dataset characteristics, cross-validation results, and comparisons between our extractors and alternative approaches.

### Pipeline Execution and Time Requirements

**Figure 2.**
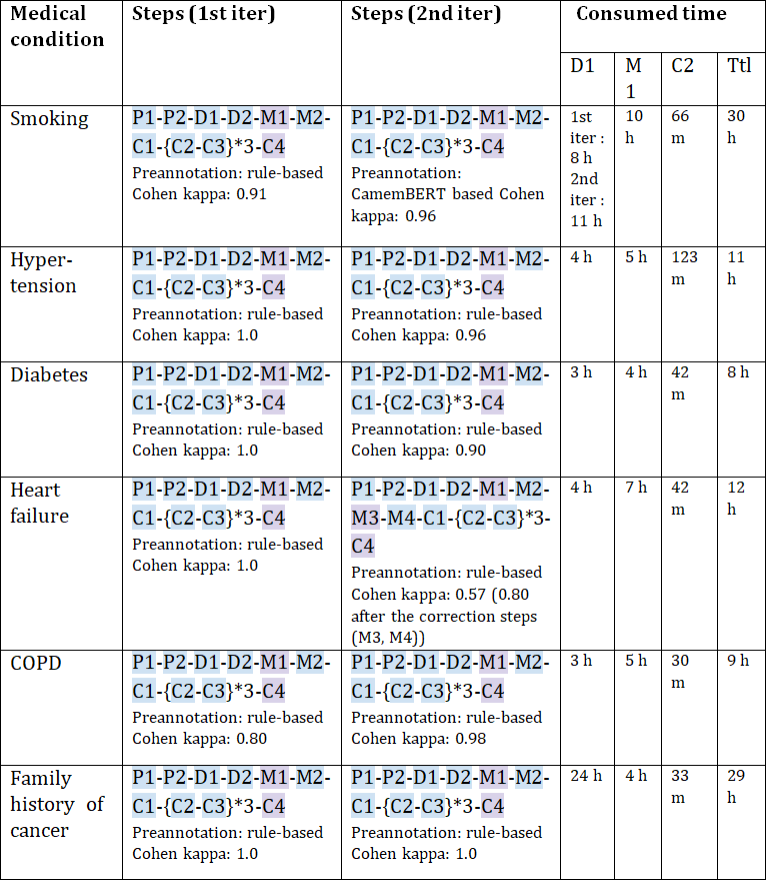
Steps in the 2 iterations of the pipeline were realized to extract each medical status. The steps within the curly braces mean that they are repeatedly realized within one iteration, and the number after the * marks how many times these steps have been repeated. The color of the steps is consistent with Figure 1; blue means automated steps and purple means manual steps. The type of model used for preannotation (PA) and the interannotator agreement score calculated at step M2 are given underneath the list of steps. The last 4 columns show the total time consumption of preannotation (D1), manual annotation (M1) during both iterations, model fine-tuning during cross-validation (C2), and the total time consumed to go through the pipeline in 2 iterations (Ttl). The time consumption is calculated by hours (h) or by minutes (m), depending on the convenience of data representation. COPD: chronic obstructive pulmonary disease.

Figure 2 details the implementation path and time consumption for each medical condition across both iterations.

Most medical conditions were preannotated using rule-based matching, except smoking status, which was preannotated in the second iteration using a CamemBERT-based extractor fine-tuned from the first, which outperformed the rules. Heart failure annotation during the second iteration initially yielded insufficient interannotator agreement (57%), necessitating disagreement analysis and correcting annotated data. The analysis revealed that the inconsistencies arose from concept confusion among annotators. A list of ambiguous terms was identified and clarified with a medical expert, incorporated into updated annotation guidelines, and used to automatically extract sentences that need revision from all annotations. These sentences were then reviewed and corrected by the annotators based on the updated guidelines.

Time requirements varied by medical condition and process step. We focused on the 3 most time-intensive steps: preannotation (D1), manual annotation (M1), and extractor training during cross-validation (C2). Preannotation (D1) speed ranged from 0.02 to 0.17 seconds per sentence, with COPD and diabetes being fastest (0.02 s/sentence) and smoking and family history of cancer slowest (0.12‐0.17 s/sentence). Manual annotation (M1) required 1.2‐2.9 seconds per sentence when considering all status labels, with diabetes and family history of cancer being the most efficient (1.2 s/sentence) and smoking being the most time-intensive (2.9 s/sentence). After removing the speed bias introduced by quickly dismissed sentences labeled as unknown, annotation speed ranged from 2.23 to 4.25 s/sentence, with hypertension being the fastest and family history of cancer the slowest, indicating that annotation speed is partly driven by sentence complexity and semantic load. Cross-validation training (C2) averaged 5‐22 min per session (of cross-validation), with COPD training being fastest (5 m/session) and hypertension slowest (22 m/session). Final extractor inference speed ranged from 0.09 to 0.12 s per sentence. Total implementation time across both iterations (Ttl) ranged from 8 hours (diabetes) to 30 hours (smoking). The time consumption and speed of all automatic steps (D1, C2, and inference of extractors) are calculated on a GPU of NVIDIA A100 40 GB graphics card.

### Training and Validation Datasets

The first iteration involved quickly preannotating 9997 medical documents and selecting 991 sentences from them for manual annotation. In order to improve extractors’ performance by training them with more medical status samples, the second iteration preannotated an additional 18,994 documents, yielding 11,128 sentences for manual annotation. Table 2 shows the distribution of medical status in sentences annotated during each iteration. The training and validation dataset for the 2nd iteration of the pipeline is the combination of all sentences annotated during both iterations (12,119 sentences in total).

**Table 2.** Number of samples of different medical status in sentences respectively annotated during the 1st (itr1) and the 2nd (itr2) iteration of the pipeline.

Medical condition	Present		Absent		Former		Unknown		Total	
itr1	itr2	itr1	itr2	itr1	itr2	itr1	itr2	itr1	itr2
Smoking	75	646	42	211	87	417	787	9854	991	11,128
Diabetes	279	257	29	37	—^a^	—	683	10,834	991	11,128
Hypertension	217	1184	8	30	—	—	766	9914	991	11,128
Heart failure	46	183	41	217	—	—	904	10,728	991	11,128
COPD^b^	22	366	0*^c^*	1*^c^*	—	—	969	10,761	991	11,128
Family history of cancer	5	2	0*^c^*	1*^c^*	—	—	986	11,125	991	11,128

a Not applicable.

b COPD: chronic obstructive pulmonary disease.

c Status with too few samples for cross-validation.

Sample sparsity was evident for certain status classifications. During the first iteration, most conditions had fewer than 300 samples per status, with absence samples typically fewer than presence samples. No samples were found for the absence of COPD and family history of cancer. We then increased preannotated documents for the second iteration, which successfully increased sample counts for most status classifications, though samples remained limited due to the absence of COPD and both the presence and absence of family history of cancer.

Among the 12,119 sentences, approximately 10% (n=1200) were randomly selected from the medical documents without preannotation, yielding an unbiased subset of 1200 sentences (unfiltered sentences [US]). We compared the distribution of medical statuses in this subset with that of the remaining 10,919 sentences (preannotated filtered sentences [PS]) selected through preannotation to assess the extent of bias introduced by this data qualification process (D1, D2).

Table 3 shows the result of this comparison. For each medical condition, we calculated the proportion for each of its statuses within US and PS, which is defined as:


(5) proportion=c(status)/c(present,absent,former)

**Table 3.** Comparison of medical status samples’ distribution (%) in 10,919 preannotated filtered sentences (PS) and 1200 unfiltered sentences (US).

Status	Smoking		Diab		Hyper		CHF^a^		COPD^b^		Fam	
US	PS	US	PS	US	PS	US	PS	US	PS	US	PS
Present	44	49	100	89	100	97.3	33.3	47.1	100	99.7	0	88
Absent	12	17	0	11	0	2.7	66.7	52.9	0	0.3	0	12
Former	44	34	—^c^	—	—	—	—	—	—	—	—	—
Prevalence of relevant statuses	0.75	13.5	0.25	5.49	0.75	13.1	0.25	4.43	0.17	3.54	0	0.07

a CHF: congestive heart failure.

b COPD: chronic obstructive pulmonary disease.

c Not applicable.

where *c*() denotes the total number of samples of one or more statuses in the set. Since the purpose of the data qualification is specifically to reduce the proportion of sentences that do not express any status information (ie, the class unknown), this category was excluded from the computation to avoid inflating the proportions of relevant statuses (ie, present, absent, and former) in the PS and obscuring the assessment of distributional changes. Instead, we report the prevalence of relevant statuses in US and PS to quantify how effectively steps D1+D2 have increased relevant samples’ density in PS by filtering out irrelevant sentences (class unknown). This measurement is defined as:


(6) $$\begin{array}{l} prevalence=c(present,absent,former)\\ /c(present,absent,former,unknown) \end{array}$$

Across most medical conditions, the distribution of status labels remained stable after the data filtering steps. The proportions of present status for smoking, diabetes, hypertension, and COPD, as well as the absent status for COPD and hypertension, showed only minor deviations between US and PS. A modest shift was observed for the former and absent statuses of smoking, whereas heart failure exhibited a more substantial change in the present status (from 33.3%, 1/3 in US to 47.1%, 228/484 in PS). Larger apparent shifts for rare categories, such as family history of cancer or the absent status of hypertension, reflect the extremely small number (or absence) of these samples in US, making baseline proportions unstable and limiting interpretability. Overall, despite shifts in certain conditions, the relative ordering of status frequencies remained consistent, suggesting preservation of the dominant patterns. Finally, the prevalence values show that the filtering steps substantially increased the density of relevant samples. For most conditions, the proportion of samples in PS was more than 10 times higher than in US (eg, from 0.75%, 9/1200 to 13.45%, 1469/10,919 for smoking, and from 0.25%, 3/1200 to 5.49%, 599/10,919 for diabetes), demonstrating the effectiveness of steps D1+D2 in removing irrelevant sentences while introducing only minor distributional bias.

### CamemBERT-Based Medical Status Extractors Evaluation

As a reminder, each extractor was trained in a multiclass classification setting to predict the status of a single medical condition. Evaluation metrics include precision, recall, specificity, and *F*-score for each individual status, as well as balanced accuracy and macro *F*-score aggregated across all statuses. Table 4 presents the performance metrics for the best-performing extractor of each medical condition after the second iteration. Extractors for diabetes and heart failure achieved excellent overall performance (macro *F*-scores of 99% and 96%, respectively). The COPD extractor achieved a macro *F*-score of 99%, although evaluation was limited to the present and unknown classes due to the lack of absent samples. The diabetes, hypertension, and COPD extractors exceeded 98% *F*-score for presence detection. The smoking extractor achieved balanced performance across status classifications (93%‐94% *F*-scores), while the hypertension extractor showed imbalanced performance between presence (98% *F*-score) and absence (86% *F*-score). The extractor for family history of cancer underperformed with only 80% *F*-score for presence detection.

**Table 4.** Performance of the best CamemBERT-large based extractors obtained after the cross-validation of the 2nd iteration of the pipeline. For each of the studied 6 medical conditions—smoking, diabetes (diab), hypertension (hyper), congestive heart failure (CHF), chronic obstructive pulmonary disease (COPD), and family history of cancer (fam)—the extractor is evaluated on each of its status (unknown, absent, present, or former) with 4 metrics: *F*-score, precision, recall, and specificity. The macro *F*-score and balanced accuracy are calculated on (and only on) the evaluated status.

Extractor	Smoking	Diab	Hyper	CHF	COPD	Fam
Unknown *F*-score	0.98	0.99	0.98	0.97	0.99	0.99
Unknown Precision	0.99	1.0	0.98	0.96	1.0	1.0
Unknown Recall	0.97	0.99	0.99	0.99	0.99	0.99
Unknown Specificity	0.99	1.0	0.98	0.94	1.0	1.0
Absent *F*-score	0.94**^a^**	1.0**^a^**	0.86*^b^*	0.97**^a^**	—^c^	—
Absent Precision	0.94	1.0	0.86	0.98	—	—
Absent Recall	0.93	1.0	0.85	0.96	—	—
Absent Specificity	0.99	1.0	0.99	0.99	—	—
Present *F*-score	0.93^a^	0.99^a^	0.98^a^	0.92^a^	0.99^a^	0.80^b^
Present Precision	0.91	0.99	0.98	0.96	0.98	0.67
Present Recall	0.95	1.0	0.98	0.89	1.0	1.0
Present Specificity	0.97	0.99	0.99	0.99	0.99	0.99
Former *F*-score	0.94^a^	—	—	—	—	—
Former Precision	0.93	—	—	—	—	—
Former Recall	0.94	—	—	—	—	—
Former Specificity	0.99	—	—	—	—	—
Macro *F*-score	0.95^a^	0.99^a^	0.94^a^	0.96^a^	0.99^a^	0.89^b^
Balanced accuracy	0.95	0.99	0.94	0.95	0.99	0.99

a Status (other than Unknown) whose *F*-score has surpassed 90%.

b *F*-score has not surpassed 90%.

c Not applicable.

Table 5 presents the results of the 3-fold cross-validation from both iterations. For each medical condition, we evaluated the stability of the extractors by reporting the mean and SD of the *F*-score for each status category, as well as for the overall macro *F*-score, across the cross-validation folds. All extractors demonstrated improved performance after the second iteration. After the first iteration, only the diabetes extractor achieved a macro *F*-score exceeding 90%, while by the second iteration, most condition extractors surpassed this threshold. Hypertension, heart failure, and COPD extractors showed the most substantial improvement (>30%).

**Table 5.** Result (average *F*-score, SD) of 3-fold stratified cross-validation with CamemBERT-large based extractors trained during the 1st (iter 1) and the 2nd (iter 2) iterations of the pipeline.

Medical condition	Present *F*-score, mean (SD)	Absent *F*-score, mean (SD)	Former *F*-score, mean (SD)	Unknown *F*-score, mean (SD)	Macro *F*-score, mean (SD)
Iter 1					
Smoking	0.78 (0.02)	0.70 (0.03)	0.88 (0.005)	0.99 (0.02)	0.83 (0.02)
Diabetes	0.89 (0.04)	0.99 (0.01)^a^	—^b^	0.98 (0.004)	0.95 (0.03)^a^
Hypertension	0.94 (0.002)	0 (0)	—	0.97 (0.003)	0.63 (0.003)
Heart failure	0.24 (0.03)	0.14 (0.1)	—	0.71 (0.09)	0.36 (0.05)
COPD^c^	0 (0)	—	—	0.93 (0.04)	0.31 (0.01)
Family history of cancer^d^	0 (0)	—	—	0.99 (0.003)	0.33 (0.001)
Iter 2					
Smoking	0.93 (0.008)	0.93 (0.005)	0.92 (0.01)	0.98 (0.0004)	0.94 (0.003)
Diabetes	0.98 (0.008)^a^	0.98 (0.02)^a^	—	0.98 (0.004)	0.98 (0.01)^a^
Hypertension	0.98 (0.002)^a^	0.85 (0.01)^e^	—	0.98 (0.002)	0.96 (0.009)^a^
Heart failure	0.90 (0.02)	0.95 (0.02)^a^	—	0.97 (0.003)	0.94 (0.003)
COPD	0.99 (0.002)^a^	—	—	0.99 (0.003)	0.99 (0.002)^a^
Family history of cancer^d^	0.55 (0.37)	—	—	0.99 (0.003)	0.73 (0.17)

a Average *F*-score more than 95% at detecting medical status other than Unknown (either iteration).

b Not applicable.

c COPD: chronic obstructive pulmonary disease.

d For family history of cancer, with only 7 positive samples across 2 iterations, calculating a SD of 37% for the *F*-score is statistically unstable and potentially misleading regarding the extractor’s reliability.

e Average *F*-score less than 90% after the second iteration.

Despite these improvements, the family history of cancer extractor achieved only 73% macro *F*-score with 17% instability, primarily due to poor performance in detecting presence (55% *F*-score, 37% instability). The hypertension extractor also showed suboptimal performance for absence detection (85% *F*-score, 1% instability). Due to insufficient samples, absence classification for COPD and family history of cancer could not be trained or evaluated.

To go one step deeper into the assessment of the extractor performances, a post hoc analysis according to document type was conducted. Table 6 reports the average *F*-scores with SD, obtained during three-fold cross-validation during the last iteration of the pipeline, for each medical status across the major clinical document types represented in the dataset.

**Table 6.** Average *F*-scores (SD) per medical status (present: pr, absent: ab, former: fm, and unknown: un) across clinical document types: Nursing care–Procedures (Nurs-Proc), Medical reports (Med report), Nursing care–Monitoring and vitals (Nurs-Monit), Multidisciplinary follow-up and daily notes (daily note), Imaging reports (image), Prescriptions and medication orders (Presc), Paramedical care (Paramed), Administrative and admission (Admin), Results and pathology reports (Patho), and Other.

Condition and status	Nurs-Proc	Med report	Nurs-Monit	Daily note	Image	Presc	Para-med	Admin	Patho	Other
Smoking, mean (SD)
pr	1.0 (N/A^a^)	0.95 (0.006)	—^b^	0.93 (0.02)	1.0 (N/A)	1.0 (N/A)	0.83 (0.236)	1.0 (N/A)	—	0.96 (0.064)
ab	—	0.92 (0.01)	—	1.0 (N/A)	1.0 (N/A)	—	—	1.0 (N/A)	—	—
fm	1.0 (N/A)	0.94 (0.01)	—	1.0 (N/A)	0.89 (0.1)	—	—	—	—	1.0 (N/A)
un	1.0 (N/A)	0.98 (0.004)	1.0 (N/A)	0.93 (0.016)	0.97 (0.029)	0.96 (0.064)	1.0 (N/A)	1.0 (N/A)	1.0 (N/A)	1.0 (N/A)
Diab, mean (SD)
pr	1.0 (N/A)	0.97 (0.012)	—	1.0 (N/A)	1.0 (N/A)	1.0 (N/A)	0.97 (0.044)	—	—	—
ab	—	0.97 (0.02)	—	1.0 (N/A)	—	—	—	—	—	—
un	1.0 (N/A)	0.99 (0.0)	1.0 (N/A)	1.0 (N/A)	1.0 (N/A)	1.0 (N/A)	0.93 (0.115)	1.0 (N/A)	1.0 (N/A)	1.0 (N/A)
Hyper, mean (SD)
pr	1.0 (N/A)	0.99 (0.002)	0 (N/A)	0.97 (0.011)	1.0 (N/A)	1.0 (N/A)	1.0 (N/A)	1.0 (N/A)	—	1.0 (N/A)
ab	—	0.84 (0.022)	—	1.0 (N/A)	—	—	—	—	—	—
un	1.0 (N/A)	0.98 (0.004)	0.99 (0.008)	0.94 (0.037)	1.0 (N/A)	1.0 (N/A)	1.0 (N/A)	1.0 (N/A)	1.0 (N/A)	1.0 (N/A)
CHF^c^, mean (SD)
pr	1.0 (N/A)	0.92 (0.029)	0 (N/A)	1.0 (N/A)	1.0 (N/A)	1.0 (N/A)	1.0 (N/A)	—	—	1.0 (N/A)
ab	—	0.98 (0.02)	—	0.8 (0)	1.0 (N/A)	—	—	—	—	1.0 (N/A)
un	1.0 (N/A)	0.98 (0.002)	0.99 (0.009)	0.95 (0.014)	1.0 (N/A)	1.0 (N/A)	1.0 (N/A)	1.0 (N/A)	—	1.0 (N/A)
COPD^d^, mean (SD)
pr	1.0 (N/A)	0.99 (0.002)	—	1.0 (N/A)	1.0 (N/A)	1.0 (N/A)	1.0 (N/A)	0.89 (0.192)	—	—
un	1.0 (N/A)	0.99 (0.001)	1.0 (N/A)	1.0 (N/A)	1.0 (N/A)	1.0 (N/A)	1.0 (N/A)	0.94 (0.079)	1.0 (N/A)	1.0 (N/A)
Fam, mean (SD)
pr	—	0.55 (0.37)	—	—	—	—	—	—	—	—
un	1.0 (N/A)	0.99 (0.003)	1.0 (N/A)	1.0 (N/A)	1.0 (N/A)	1.0 (N/A)	1.0 (N/A)	1.0 (N/A)	1.0 (N/A)	1.0 (N/A)

a N/A: not applicable.

b Not available.

c CHF: congestive heart failure.

d COPD: chronic obstructive pulmonary disease.

For 72 status–document-type combinations, no sample was retrieved in validation sets, and performance cannot be discussed (marked with an “-”). For the remaining 115 status document-type combinations, we can divide results into 2 categories.

Those with comparable performance (less than 5% decrease) or improvement on specific documents (109/115, 94.8%) and those where a decrease is observed (6/115, 5.2%). In the first category, 79 instances show a score of 1.0 +/– N/A, indicating that only one-fold contains content for evaluation. This perfect performance also suggests that few samples were available for evaluation. In the second category, 2 instances were only evaluated in one fold, and extractors missed the status (ie, for hypertension presence and heart failure presence in the nurse report). For the 4 others (smoking presence and diabetes indifference in paramedical report, absence of heart failure in daily note, and presence of COPD in administration note), a decrease is observed, but performance remains acceptable (>80%). Examination of false positives and false negatives in these 4 cases suggests that most errors stem from the misleading narrative style of clinical notes. For example, in a paramedical report, “OH chronique sevré tabac syndrome dépressif” (Chronic alcohol use weaned tobacco use depressive disorder) was misclassified as former smoking because the boundary between alcohol withdrawal and smoking was unclear. Another sentence, “des atcd cardio,HTA,DT2non traité*”* (A history of cardiac disease, untreated hypertension, and type 2 diabetes) was misclassified as absence of diabetes because the negation “non” was attached to the diabetes “DT2” rather than to the treatment “traité*.*” The extractor also produces some persistent errors due to the medical complexity of the content, which is challenging even for specialist reviewers; for instance, a sentence in a daily note was misclassified as absent of heart failure because it contains this expression “avec une fraction d'éjection ventriculaire conservée” (with a preserved ventricular ejection fraction), which does not exclude entirely the possibility of having a heart failure. In conclusion, although the representation of entities is very sparse, we observe overall good robustness of the extractors depending on the type of document encountered. To go further, the number of annotated sentences would need to be increased.

### Comparison With Alternative Approaches

**Figure 3.**
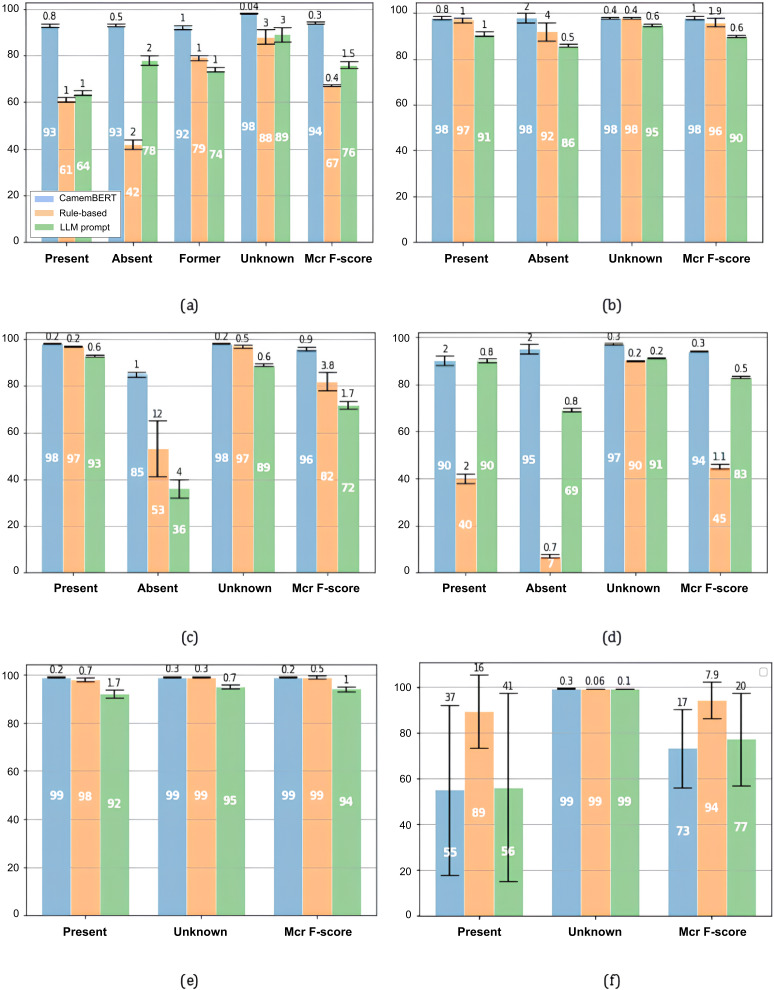
Comparison of the performance of extractors based on 3 different approaches: fine-tuned CamemBERT, (A) rule-based extraction and large language model prompt query for smoking, (B) diabetes, (C) hypertension, (D) heart failure, (E) chronic obstructive pulmonary disease, and (F) family history of cancer. The white number on each bar marks the average macro *F*-score percentage, and the number above each bar represents the SD (also percentage) calculated from cross-validation. The performance is evaluated by testing all extractors on the same validation datasets used during the 3-fold cross-validation of the 2nd iteration of the pipeline.

Figure 3 presents a comparison of the extractors developed using CamemBERT fine-tuning, rule-based methods, and LLM prompting (Mixtral-8×7B-v0.1). As a reminder, all extractors were evaluated on the same validation sets used for the 3-fold cross-validation of CamemBERT-based extractors. For conditions with ample annotated examples, such as smoking and heart failure, CamemBERT-based extractors achieved the highest performance (also confirmed by paired *t* test analysis; Multimedia Appendix 3). In contrast, for conditions with limited training data, rule-based extractors performed comparably to or better than CamemBERT-based models, reflecting their robustness in low-data scenarios. This was particularly evident for family history of cancer, where CamemBERT-based extractors achieved an average macro *F*-score of 0.73 with a cross-validation SD of 17%, while the rule-based approach reached an average macro *F*-score of 0.94 with a substantially lower SD of 7.9%.

Compared with CamemBERT-based and rule-based extractors, LLM-prompted extractors showed intermediate performance overall but were less efficient in our setting. Using Mixtral-8×7B-v0.1, sentence-level extraction required 12‐24 seconds per sentence and 35 GB of GPU memory, which is slower and more resource-intensive than CamemBERT (0.09‐0.12 s per sentence; 2 GB GPU).

These findings indicate that CamemBERT-based and rule-based methods are the most suitable approaches for our institutional resource constraints, and that no single extraction approach is universally optimal across all clinical scenarios.

## Discussion

### Overview

This section examines the advantages and limitations of the MSEP pipeline within our institutional setting and explores how its design can support local development of clinical NLP extractors.

### Technical Advantages

#### Efficiency Gains for Clinical Text Annotation

The MSEP pipeline addresses challenges posed by the low density of medical-status information in large institutional corpora, which can hinder the efficient local development of information extraction systems. Its preannotation step helps prioritize sentences with potentially relevant information, and reframing extraction as sentence classification allows annotators to make quicker labeling decisions. Together, these components streamline dataset construction and reduce the operational burden of annotation.

Although our annotators were unavailable for extensive medical entity annotation, preventing a direct comparison of annotation speed in our institution, prior work has shown that sentence classification generally requires less annotation time and effort than word-level NER [57-60]. This is expected, as sentence classification involves assigning a single label to an entire sentence, whereas NER requires annotators to identify multiple entities within a sentence and decide on their boundaries, increasing the complexity and time required for annotation [61]. This improvement in annotation speed is also supported by an empirical comparison with a previous study on NER efficiency [20], as our manual sentence classification ranged from 1.2 to 2.9 seconds per sentence (or 2.23 to 4.25 s per sentence when considering only informative sentences), significantly faster than the 7.82 to 16.48 s reported for NER.

The sentence classification-based extractors also demonstrated faster inference speed than the traditional composite extractors (NER + qualifier). In our study, extractors of rule-based NER combined with EDS-NLP qualifiers were tested for smoking and family history of cancer during the preannotation step of the first iteration of the pipeline. The composite approach (0.17 s/sentence) was notably slower than our CamemBERT-based extractor (0.09‐0.12 s/sentence) and rules-only preannotation models (0.02‐0.04 s/sentence). Importantly, this efficiency gain did not compromise performance. Compared with recent medical status extractors, our pipeline achieved comparable or superior results. Our extractors reached *F*-scores of 99%, 95%, 96%, and 99% for diabetes, smoking, heart failure, and COPD, respectively, matching or exceeding performance metrics reported in recent literature [16].

#### Hybrid, Configurable, and Locally Deployable Pipeline Architecture

The MSEP pipeline was designed as a flexible and configurable framework that enables the local deployment of hybrid extraction approaches across diverse institutional settings. The accompanying Python package (repository link in Multimedia Appendix 1) exposes all key components, such as preannotation models (D1), sentence filtering criteria (D2), cross-validation strategy (C2), and extractor choice, as configurable arguments. This modular design allows researchers to tailor each step of the workflow, including the choice of extraction approach, according to their resource constraints, characteristics of the targeted information, and institutional requirements. For instance, in our experiments, fine-tuned CamemBERT models produced satisfactory performance for most medical statuses, whereas rule-based extractors yielded more stable results for conditions with limited representation in the dataset. This means that the pipeline can be configured to favor rule-based extraction in low-example scenarios. Conversely, in settings with limited annotation resources or domain expertise, the pipeline can be configured to generate LLM prompt-based extractors, which can be readily improved by providing more detailed instructions and examples in the prompts [62].

Our institutional experiments further illustrated the practical feasibility of deploying MSEP within a local clinical setting. First, the full workflow, including preannotation, manual validation, model training, and validation, was applied independently to 6 medical conditions and completed within 99 hours in total (approximately 16.5 h per condition). This was achieved with 2 annotators and a single NVIDIA A100 40 GB GPU, a setup feasible for many medical research institutions. Second, the models were trained and evaluated on a heterogeneous set of clinical documents (including hospital discharge summaries, consultation notes, and reports from multiple specialties). The comparable performance observed across these diverse sources indicates that the workflow can be operationalized effectively within diverse documentation practices at a single institution.

While our current evaluation remains monocentric, MSEP’s packaging and explicit parameterization are designed to facilitate reproducible deployment in other institutions. Applying identical configurations on comparable data would be expected to yield consistent results, and extending the approach to multicentric validation represents an important next step toward confirming generalizability and cross-site reproducibility.

#### Enhanced Monitoring and Optimization Mechanic

The pipeline provides means for evaluating and improving each of its mean processes (data qualification, manual annotation and correction, model training, and cross-validation). Preannotation (D1) allows estimation and supervision on corpus’ sample density and medical status’ distribution, which can be adjusted by sentence filtering (D2); interannotator agreement calculation (M2) and analysis of annotation disagreements (M3) not only revise the quality of annotated sentences, but also reveal problems in annotation guidelines and improve them accordingly (M4); extractors’ assessment and comparison (C4) enables comprehensive model evaluation and informs decisions on the need for and direction of further improvements.

The iterative nature of our pipeline provides a means for monitoring and optimizing dataset size. By accumulating annotated data gradually and evaluating extractors with cross-validation at each iteration, we can determine when to halt manual annotation, limiting resource expenditure to reasonable levels. We also considered active learning as an alternative approach, where the model selects samples difficult to predict for prioritized manual annotation and updates itself with the received annotation [63]. In theory, this approach can maximize annotation efficiency by prioritizing the most informative samples for model improvement. However, we ultimately rejected it because model updates were disproportionately influenced by status categories with the most samples, and uncertain sample distribution prevented effective loss function weighting. Nevertheless, active learning remains valuable when balanced datasets are available or when sample balancing can be implemented during data acquisition [64].

### Importance for Clinical Research

#### Response to Clinical Text-Mining Challenges

Clinical text mining remains challenging due to the unstructured and context-dependent nature of medical narratives. Our evaluation shows that sentence-level classification, as implemented in MSEP, effectively addresses several of these challenges in practice. The approach demonstrated high accuracy across heterogeneous document types, confirming that the model captures contextual cues, such as negation or temporality, without relying on context qualifiers. Compared with traditional NER-based systems, which require token-level annotation and post hoc context detection, the sentence classification strategy substantially reduces annotation time and complexity. The high *F*-scores obtained for conditions such as smoking and diabetes illustrate that this formulation aligns well with the way medical status is expressed, which is typically at the sentence level rather than through isolated entities.

To our knowledge, there is currently no existing pipeline in the clinical NLP literature that applies sentence classification for medical status extraction. While sentence classification has been explored in other domains, such as sentiment analysis or social media mining, its application to clinical narratives remains underdeveloped and has not yet been systematically assessed for this purpose.

#### Easy Implementation as a Research Tool for Clinical Studies

Clinical text-mining efforts are often constrained by privacy regulations and institutional heterogeneity that prevent direct data or model sharing across sites [16,19,36-38]. The MSEP framework was designed to address these issues by enabling efficient local dataset construction, providing flexible configuration of extraction approaches according to institutional constraints, and supporting controlled resource expenditure through performance-monitored iteration.

Beyond initial model development, MSEP demonstrated utility as an upstream component in broader research workflows that can support more specialized downstream tasks. When applied first, MSEP extractors can identify only the documents or sentences that contain the targeted medical status, allowing researchers to exclude irrelevant material entirely and thus avoid expending effort on processing noninformative documents. In practice, this functionality has already been demonstrated within our institution, where a smoking-status classifier created by MSEP was applied to an independent corpus of 300 clinical reports, efficiently filtering for documents concerning patients who were smokers and achieving an *F*-score of 99.6% for detecting smoking-related content. Downstream tasks, such as extracting smoking duration or tobacco brand, can then be focused solely on these filtered documents, reducing workload and improving overall efficiency.

### Current Limitations

Our study has several limitations worth noting. First, due to the availability of medical experts, we did not conduct a direct comparison between sentence classification and NER. Instead, we relied on empirical observations and general consensus to suggest that sentence classification offers greater annotation efficiency. While the improvement in annotation speed appears supported, the impact on annotation quality remains to be verified.

Second, clinical document type is a factor that can influence the interpretation of medical status occurrence in text, which was not fully addressed in our study. For instance, a mention of “hypertension” in a medical report, especially within the “Past Medical History” section, typically refers to a chronic condition. In contrast, if hypertension is noted in daily progress notes, it is more likely to reflect an acute episode under current observation or management. Therefore, when applying MSEP to clinical documents from a CDW, it may be necessary to incorporate a document selection step during data collection (P1), not only to include relevant document types, but also to focus on specific sections within documents, such as the “Past Medical History” section, when these are more likely to align with the study objectives.

Third, the construction and usage of the datasets are subject to several sources of bias. Preannotation and filtering were used to increase the density of informative samples in the dataset, but mislabeling by preannotation rules could have led to the exclusion of relevant sentences (eg, those labeled as “unknown”). To assess this risk, 10% of our dataset consisted of nonpreannotated sentences, which were used to evaluate potential bias introduced by the preannotation rules. While most conditions showed limited distortion, a notable shift was observed for the present status of heart failure, underscoring that preannotation can influence class distributions. A further concern is the risk of circular bias, which may be induced by using sentences initially selected by rules to evaluate those same rule-based extractors. To limit this risk, we constructed the annotated corpus by combining the preannotations from all descriptors rather than relying on a single rule-based extractor per condition, resulting in a relatively small proportion of validation sentences originating from the same rule set (eg, 3.4%‐3.6% for COPD). Evaluating extractors is a complex task in such large datasets. Ideally, most of the dataset would need to be annotated to determine actual performance. However, this task requires too much annotation time for most noninformative sentences. Our approach to selection by multiple preannotations allows us to limit this time while verifying that little bias appears to be induced (eg, confirmed using sentences without preannotation). Finally, although identical sentences were deduplicated before cross-validation, dataset partitioning was not stratified by Patient_ID. As a result, sentences about the same patient could appear across validation folds, introducing a potential risk of data leakage. The proportion of validation sentences with patient overlap ranged from 3% to 8%. To assess the impact of this overlap, we revalidated the models after removing these sentences from the validation sets. Performance changes were minimal (less than 1% of drop for macro *F*-score of each cross-validation fold), suggesting that patient-level overlap did not artificially inflate extractor performance. Moving forward, these extractors should be used on other datasets while keeping in mind their potential limitations.

Fourth, the evaluation of certain medical statuses was limited by their very low prevalence in the corpus. For COPD and family history of cancer, the Absent status was represented by only a single instance, preventing meaningful assessment of the extractor’s ability to distinguish absent from unknown. Consequently, both extractors effectively functioned as binary classifiers (present vs unknown). This limitation reflects typical documentation practices: the absence of a specific condition (eg, COPD) is rarely stated explicitly, as doing so would require exhaustively listing all conditions that are not present; the issue is somewhat different for family history of cancer, where the absence of this condition is generally more informative (“no family history of cancer”) and therefore more likely to be mentioned, although such mentions remained infrequent in our dataset.

Finally, decoder-only LLMs were not fully used due to initial infrastructure limitations. Our computing environment, a secure server at the University Hospital of Rennes with 112 Intel(R) Xeon(R) Gold 6258R CPU cores and limited access to an NVIDIA A100 40 GB GPU, was insufficient for efficient, prompt-based inference (12‐24 s/sentence when performing medical status extraction) or fine-tuning (requiring ≥64 GB VRAM).

### Perspectives and Future Applications

Beyond its current implementation, the pipeline can be extended to broader medical information extraction tasks. Ongoing work includes detecting additional statuses relevant to bladder cancer [3] and extracting finer-grained information such as smoking duration and cigarette type. By first classifying sentences containing smoking mentions, the pipeline narrows the scope for subsequent extractions, improving efficiency.

Future studies will also address current limitations. First, a direct comparison of annotation speed and quality between sentence classification and NER will provide stronger evidence for our methodological choice. Second, it is well known that medical documents contain information, structures, and sometimes vocabulary that are unique to them. For our specific study, we have demonstrated a certain robustness of extractors to these phenomena. To reinforce this finding, more instances will need to be annotated, bearing in mind that we are working in a very sparse context. Third, synthetic datasets will be explored to mitigate preannotation bias, balance category distributions, and reduce reliance on sensitive records. They may also improve extractor performance on medical conditions with scarce samples (eg, family history of cancer). Prior studies have investigated GANs for generating synthetic structured EHRs [40]; while ensuring both utility and confidentiality remain challenging, synthetic data offer a promising path forward for clinical NLP.

Finally, the Ollama framework [65] will be used for a better exploration of LLM prompt potential. This framework enables accelerated local inference, improving medical status annotation speed with Mixtral-8x7B-v0.1 to 0.2 seconds per sentence. Although LLM prompting has not yet reached the same level of performance as CamemBERT fine-tuning for certain conditions, further exploration of prompt design and fine-tuning strategies may be pursued as infrastructure improves. However, it is important to keep in mind the computational and ecological cost of such a strategy when rule-based and CamemBERT-based approaches can provide satisfactory results.

Together, these perspectives aim to strengthen the robustness, reproducibility, and applicability of the pipeline across diverse clinical research settings.

### Conclusions

We introduced MSEP, a modular and hybrid methodological framework for extracting medical status information from unstructured clinical text using sentence classification. In our institutional case study, the pipeline substantially reduced annotation effort: sentence-level labeling with preannotation required only 1.2‐2.9 seconds per sentence. MSEP successfully supported the implementation of 3 extraction approaches: rule-based methods, fine-tuned CamemBERT model, and LLM prompting, each suited to different data and resource scenarios. Fine-tuned CamemBERT model achieved high performance for conditions with sufficient training examples (macro *F*-score>94%), whereas rule-based methods provided more stable results for sparsely represented conditions. Together, these findings highlight MSEP’s value as a research tool that accelerates local dataset creation and enables flexible deployment of extraction systems. Importantly, all components of the pipeline are distributed as a ready-to-use Python package.

## Supplementary material

10.2196/77409 Multimedia Appendix 1 Pipeline configuration demonstration.

10.2196/77409 Multimedia Appendix 2 Annotation guidelines.

10.2196/77409 Multimedia Appendix 3 Complementary paired t-test evaluation of extractor performance across cross-validation folds.

## Abbreviations

CDW: clinical data warehouse
COPD: chronic obstructive pulmonary disease
LLM: large language model
MSEP: Medical Status Extraction Pipeline
n2c2: National NLP Clinical Challenges
NER: named entity recognition
NLP: natural language processing
PS: preannotated filtered sentences
US: unfiltered sentences
