# Supplementary material for: A Sentence Classification–Based Medical Status Extraction Pipeline for Electronic Health Records: Institutional Case Study
Source: JMIR Med Inform. 2026 Mar 26;14:e77409. doi: 10.2196/77409 (PMC13044345; doi:10.2196/77409)
Supplement: Multimedia Appendix 1 [file medinform-v14-e77409-s001.docx]

## Multimedia Appendix 1

**Pipeline configuration demonstration**

The MSEP is highly configurable. Each step in MSEP has one or more parameters to customize according to the need of different studies. The functions and modules constructed to realize the steps in the pipeline are compiled as a python package (package *msep*) available at <https://gitlab.com/ricdc/outils/msep>. Table A2 shows the parameters of each step in MSEP, their definition and value defined in our study for extracting statuses of 6 medical conditions, as well as their setting with the *msep* package.

Table S1: Demonstration of the MSEP pipeline configuration using example values from this study.

| **Step** | **Parameter name** | **Definition** | **Value in this study** | **Configuration with *msep* package** |
| --- | --- | --- | --- | --- |
| **P1** | data_source | The source of unstructured medical texts | ehop data warehouse | - |
| **P1** | criterion_for_ document_selection | The criterion for selecting documents for dataset construction | randomly select a number of documents that contains no more than 360 000 sentences to avoid overlong pre-annotation | - |
| **P2** | sentence_segmenter | The tool used for sentence segmentation | [spaCy](https://spacy.io/) model [*fr_core_news_md*](https://spacy.io/models/fr#fr_core_news_md) | set value to argument *segmenter_model* of function *msep.preprocessing.sentence_segmentation()* |
| **D1** | source_of_medical_ knowledge | The source of medical knowledge | medical specialists | - |
| **D1** | pre-annotation_ model | The model used for pre-annotation | manually crafted rules | call the rule-based pre-annotation functions in module *msep.preannotation_rules* |
| **D2** | criterion_for_ sentence_selection | The criterion for selecting pre-annotated sentence for manual annotation | 100% of the sentences with pre-annotated as medical status samples + 10  % of the sentences without any medical status pre-annotated + 1 000 non pre-annotated sentences; sample = sentence annotated with a present/absent/former status of a medical condition | *msep.data_selection.sample_sentence_selection()* function allows sentence selection; define selection mode (by proportion/number of different statuses) in argument *selecting_function*; define the exact selecting proportion/number for each status in argument *category_selection_ratio* |
| **M1** | annotation_tool | The tool used for manual annotation | [prodigy](https://prodi.gy/) annotation interface | - (the package is designed for default compatibility with the Prodigy interface, use *msep.manual_annotation_utils.prodigy_input_generator()* to generate prodigy input file) |
| **M1** | annotation_strategy | The arrangement of manual annotation for annotators, specifying the number of annotators and how they cooperate | 2 annotators, 90% content annotated separately, 10% annotated commonly | - |
| **M2** | agreement_metric_ and_threshold | The metric used to measure the inter-annotator agreement, and the threshold for a perfect agreement | [Cohen’s kappa coefficient;](https://scikit-learn.org/stable/modules/generated/sklearn.metrics.cohen_kappa_score.html) 80% | call *msep.manual_annotation_utils.cohen_kappa_annotator_agreement()* function to calculate the [Cohen’s kappa coefficient](https://scikit-learn.org/stable/modules/generated/sklearn.metrics.cohen_kappa_score.html) between annotations given by different annotators |
| **M3** | analysis_method | The method for analysing the disagreements between the annotators | manually observe the disagreements between annotators and find the reason of disagreements | - (*msep.manual_annotation_utils.cohen_ kappa_annotator_agreement()* function returns also a list of annotation disagreements that aids this analysis) |
| **M4** | correcting_method | The method chosen for correcting the annotation disagreements | manual correction assisted by terminology-based rules developed in consultation with medical experts | the function *msep.manual_annotation_utils.correction()* assists the manual resolution of annotation disagreements by identifying sentences that may contain terminology to be added or deleted; arguments *ajout* and *supprime* take respectively a list of terms to be added or to be deleted |
| **C1** | grouping_criteria | The partition strategy and the criteria for grouping training & validating datasets for stratified cross validation | annotated data randomly split into 3 datasets with same size and medical status sample proportion, from which 2 are combined as the training set, and the remaining one serves as the validation set | function *msep.cross_validation.stratified_data_split()* allows random and balanced data partition; argument *n* defines number of data groups |
| **C2** | neural_network_ type_and_model_ name | The deep neural network type and the specific name of the deep learning model chosen for the cross validation | transformer; [camembert-large](https://huggingface.co/almanach/camembert-large) | function *dataset_formatting_for_finetuning()* formats annotated data into training datasets compatible with pre-trained transformer-based language model; define the model to be trained/fine-tuned in the argument *path_to_pretrained_model* |
| **C2** | set_of_ hyperparameters | The hyperparameters for the deep learning process | learning rate = 2e-5, batch size = 5, max number of epoch = 15, early stopping patience = 5, loss function = binary cross entropy (with rescaled weight given to the loss of each status of a medical condition) | - (customize your training session with python’s *transformers.Trainer* class) |
| **C3** | evaluation_metrics | The metrics chosen to evaluate medical status extraction result for all approaches | on each status : precision, recall, specificity, f-score; on all status : macro F-score, balanced accuracy; on all evaluation scores : standard deviation (between different extractors for the same medical status) | function *msep.cross_validation.compute_metrics()* evaluates the precision, recall, specificity, f-score of an extractor for single status extraction, and macro F-score, balanced accuracy for all status extraction |
| **C4** | assessment_method | The method for assessing the extractors obtained after an iteration of the pipeline in order to decide whether further improvement is needed | evaluate concurrent models on validating datasets by applying the same evaluation metrics and compare the result with our chosen model | - |
| **C4** | concurrent_model | Models used to compare with the extractors constructed using the pipeline | Mixtral-8×7B-v0.1[,](https://huggingface.co/mistralai/Mixtral-8x7B-v0.1)  manually crafted rules | function *msep.concurrent_LLM_extractors. llm_annotate()* allows testing LLM prompts with a defined (decoder-only) LLM in the argument path_to_llm and predefined prompt in the argument *prompt_template_of_your_choice* |

**Detailed configuration explanation with the example of our study**

For the step Collect Unstructured Texts (**P1**), the parameter *data_source* is defined that indicates the source of unstructured data to be processed by the pipeline. In this study, the *data_source* consists of 799 470 documents of 5398 patients extracted from the Clinical Data Warehouse of the Rennes University Hospital. Additionally, *criterion_for_ document_selection* is defined to select documents for dataset creation. This criterion can be a random selection to simply reduce document number, or a selection based on research objectives, such as medical document type. In this study, 28 991 documents were randomly selected to create the training and validating dataset (9 997 documents containing 164 572 sentences for the first iteration of the pipeline, 18 994 documents containing 351 494 sentences for the second iteration). We chose these numbers of documents to limit the time needed for pre-annotation (**D1**).

At step Segment into Sentences (**P2**), parameter *sentence_segmenter* is defined to indicate the model used for segmenting the text in chosen documents into sentences. In this study we used a spaCy model fr_core_news_md which has been considered as the most suitable model for segmenting our documents after comparison with the sentence segmenters of [NLTK](https://www.nltk.org/) and [EDS-NLP](https://aphp.github.io/edsnlp/latest/). The module *preprocessing* in package *msep* can be used to perform sentence segmentation implementing spaCy models. Additionally, other processes can be added to this step in order to prepare the data for further manipulation, such as text cleaning and text parsing.

For pre-annotation (**D1**), parameter *pre-annotation_model* is defined to indicate models or approaches used to pre-annotate texts, and if they are based on manually crafted rules, parameter *source_of_medical_ knowledge* is defined to indicate the source of knowledge used for the construction of rules. This step can be realized using the module *preannotation_rules* in package *msep*, which calls the rules defined in our study for extracting status of the six medical conditions. In this study, the pre-annotation rules were based on medical knowledge, in particular from keyword lists, provided by specialists. The rules match patterns in a sentence that correspond to a confirmation or a negation of a medical condition’s presence, and annotate the sentence with a corresponding label. Different from other medical conditions, the pre-annotation for smoking and family history of cancer were realized by combining keyword matching rules with a rule-based EDS-NLP qualifier that characterizes negation and family member relevancy in a sentence. Since keyword matching rules work in the same way as a rule-based NER model, this allowed us to compare the inference speed of our extractors with composite extractors combining NER model and qualifier. From the second iteration, depending on the performance, either the rules or the extractors obtained during the previous iteration were applied to perform the pre-annotation.

The step Filter Sentences (**D2**) aims to focus manual annotation by choosing relevant sentences and therefore counteracts sample sparsity in the datasets. This step requires defining the parameter *criterion_for_sentence_selection* which specifies the criteria for sentence selection that should be coherent with the objectives of study. The module *data_selection* in package *msep* helps realising this step which provides functions that allow selecting sentences by proportion or number of annotated medical status. In this study, we assumed that sentences with medical status identified by the pre-annotation are more likely to be a sample of medical status than those without. Our criteria involve selecting all sentences with at least one medical status automatically pre-annotated (11 019 sentences, 900 for the first iteration and 10 119 for the second), and adding 10% sentences without pre-annotation (1 100 sentences, 91 for the first iteration and 1009 for the second), leading to a total of 12 119 sentences to be manually reviewed.

The step Annotation by Medical Specialists (**M1**) requires defining *annotation_tool* and *annotation_strategy*. The former defines the tool used to perform manual annotation, the later guides the annotation process in coherence with research objectives. In this study, manual annotations were performed by two experts, using the Prodigy [annotation interface](https://prodi.gy/). This tool was retained since it allows simultaneous annotation by multiple persons. Our annotation strategy involves half of the pre-annotated sentences presented to each annotator and 1000 sentences randomly selected and shown to both experts for common annotation. Commonly annotated sentences were used to ensure agreement between annotators. Inter-annotator agreement was measured at step Calculate Inter-annotator Agreement (**M2**). The parameter *agreement_metric_and_threshold* should be defined at this step that indicates the appropriate metrics for inter-annotator agreement calculation, and the threshold for agreement. In this study we measured inter-annotator agreement by calculating the Cohen’s kappa coefficient, and we set the threshold of agreement to 80%. If the coefficient were above 80%, their annotation was considered as homogeneous; if not, the annotation was considered as conflictual and correction strategies were leveraged. Disagreements between the annotators were analyzed at step Disagreement Analysis (**M3**), with the parameter *analysis_method* indicating the chosen method for analysing annotation disagreements. This analysis guides the correction of annotation disagreements at step Annotation Correction (**M4**), which requires defining the method for this correction in parameter *correcting_method*. In this study, the annotation disagreements were manually analyzed and corrected by consulting an external medical specialist which settled disagreements. The annotation guidelines were improved accordingly. As for the method of Annotation Correction, we developed rules based on annotation disagreement analysis that were applied to the entire annotated dataset to automatically identify sentences that potentially need correction. Then, the annotators manually revised these sentences based on the new annotation guidelines. The module *manual_annotation_utils* of package *msep* contains functions that allow transforming pre-annotated sentences to prodigy input file format, calculating inter-annotator agreement and applying correction rules on annotated sentences.

Figure S1 shows how data flowed in the MSEP pipeline implemented in our study. The figure focus on the data flow from step P1 to M1, after which the datasets construction was completed.

Figure A2: Flowchart of data processed in our study


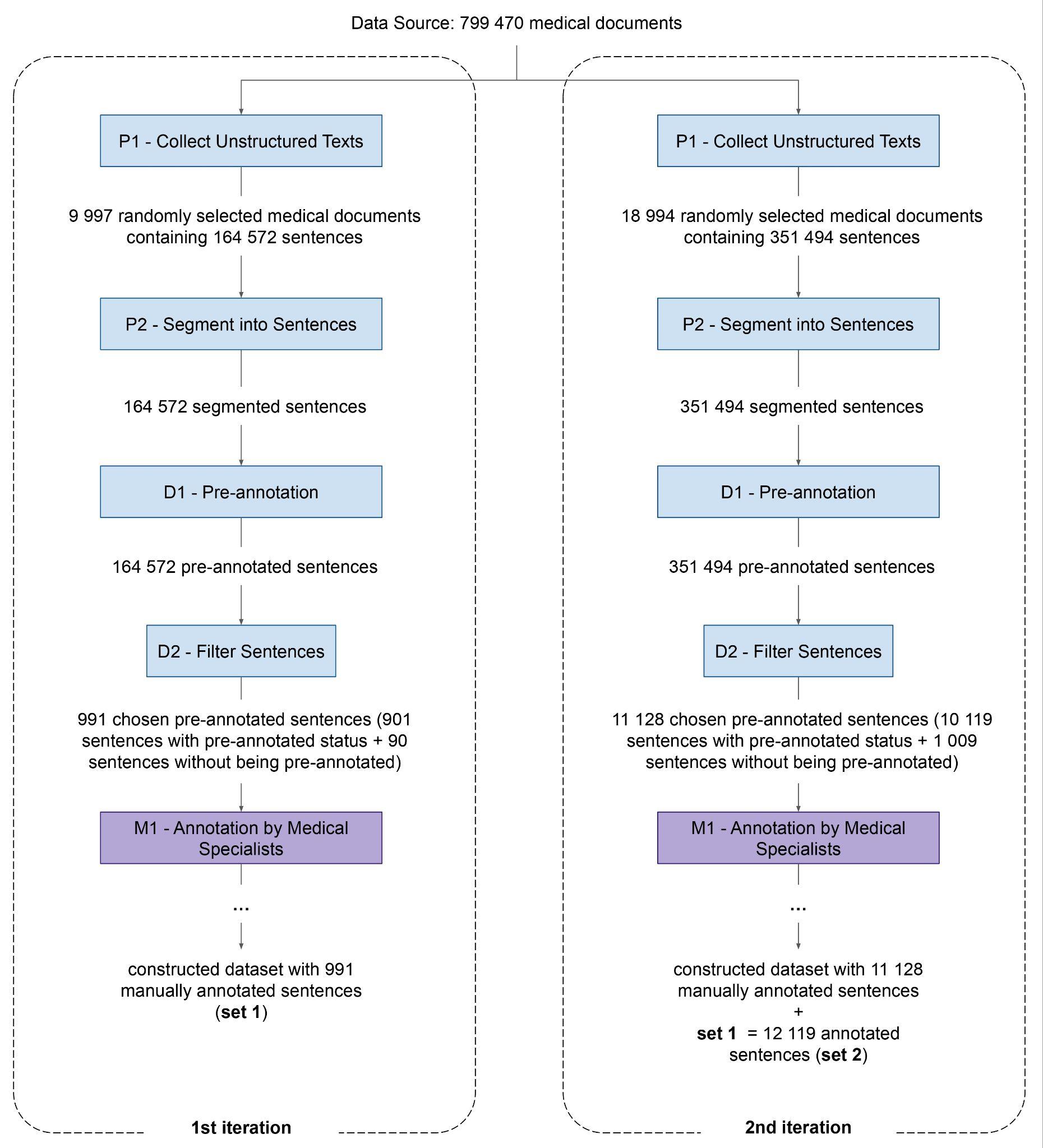


Once the annotated datasets are created, the cross-validation begins from step Split Data into N Groups (**C1**). The parameter *grouping_criteria* is defined, which specifies the number of data groups to form and the criteria based on which they should be separated. In this study, we randomly split the annotated data into 3 sets with equal size to conduct a 3-fold stratified cross-validation. Our cross-validation consists therefore of 3 sessions. In order to guarantee that all status of a medical condition can be learned and tested, all of the 3 data groups contain the same number of samples for each status. We choose to do 3-fold cross validation instead of using the classic 10-fold strategy because on one hand this shortens the time required by the training process, on the other hand the validating dataset contains more instances of medical status and thus increases the credibility of the result.

For each session, an extractor is obtained by fine-tuning/training a deep learning model (**C2**), which is then evaluated (**C3**). For step **C2**, the type and name of the deep model used for training/finetuning the extractor is defined in parameter *neural_network_type_and_model_name*, and the configuration of the deep model for training is set in the parameter *set_of_hyperparameters*. For step **C3**, the metrics used for evaluating the extractors are defined in the parameter *evaluation_metrics*. In this study, our chosen deep learning model was a CamemBERT model called camembert-large. We preferred this model as it is a French language model based on a transformer neural network pre-trained on robust French corpus, so it can achieve high performance via simple fine-tuning. We implemented the [CamembertForSequenceClassification](https://huggingface.co/docs/transformers/model_doc/camembert#transformers.CamembertForSequenceClassification) module to minimize the work of configuring the neural network for multi-label classification tasks, with some hyperparameters manually set (see table A2 for more details). The loss function was weighted according to the proportion of a medical condition’s different status to avoid unbalanced classification that favors status that have over-numbered samples in the training datasets. For each cross-validation session, performance of the trained extractor was evaluated on precision, recall, specificity, f-score for individual status, and balanced accuracy and macro f-score computed for overall assessment. These metrics reflect the performance of the extractor from different aspects without being biased about their proportion in the dataset. For each metric, average and standard deviation were calculated to assess the stability of the extractor.

The module *cross_validation* of the package *msep* contains functions for data groups splitting, formatting input data for training sessions and evaluating the trained models. And the module *CamemBERT_medical_status_extractors* provide functions that call the trained extractors to annotate sentences individually or iteratively.

In order to enrich the perspective of the pipeline’s evaluation, the performance of constructed extractors are compared with other approaches of medical status extraction at step Performance Comparison and Assessment (**C4**). Two parameters are defined at this step: *assessment_method* and *concurrent_model*. The former indicates how the extractors’ performance is compared and assessed, and the latter specifies the type, name and approach of the models to be compared with the trained extractors. In this study, the performance of all models is compared and analysed manually. For the comparison, the rule-based and the LLM prompts based extractors were evaluated on the same validating sets used during the cross-validation of the 2nd iteration of the pipeline. The rule-based extractors use the same rules implemented in the pipeline for pre-annotating the medical documents The LLM that we tested for medical status extraction is called [Mixtral-8x7B-v0.1](https://huggingface.co/mistralai/Mixtral-8x7B-v0.1), a powerful and robust language model developed by the Mistral AI team. It has been tested and applied for many NLP tasks, especially for text generation, and has achieved high performance without fine-tuning. In order to extract medical status using Mistral’s text generation function, we have designed prompts that define different status of medical conditions to be extracted and demand the model to generate corresponding labels to sentences in validating datasets. The prompts are designed based on the annotation guidelines and the keyword lists used for rule-based pre-annotation to ensure a fair comparison with the CamemBERT based and rule-based extractors. The module *concurrent_LLM_extractors* of the package *msep* contains the designed LLM prompts and provides a function that calls them to annotate sentences.
