# Supplementary material for: A Sentence Classification–Based Medical Status Extraction Pipeline for Electronic Health Records: Institutional Case Study
Source: JMIR Med Inform. 2026 Mar 26;14:e77409. doi: 10.2196/77409 (PMC13044345; doi:10.2196/77409)
Supplement: Multimedia Appendix 2 [file medinform-v14-e77409-s002.docx]

## Multimedia Appendix 2

**Annotation guidelines**

## *Objective and general principles*

We annotate the status of 6 medical conditions: smoking, diabetes, hypertension, heart failure, COPD and family history of cancer. Annotators annotate the status of one and only one medical condition at a time. The annotation is performed by giving the corresponding label to the whole sentence. Different statuses of a medical condition are mutually exclusive. When annotating the medical status of a medical condition, the sentence receives at most one label.

## *Annotation content definition*

Table S1: Definition of targeted medical status and their label

| **Medical Condition** | | **Status** | **Label** | **Description** |
| --- | --- | --- | --- | --- |
| smoking | | present | smoking_present | The sentence indicates or implies that the patient is an active smoker. |
|  |  | absent | smoking_absent | The sentence indicates or implies that the patient does not smoke. |
|  |  | former | smoking_former | The sentence indicates or implies that the patient used to smoke and has stopped now. |
|  |  | unknown | smoking_unknown | The sentence does not contain any information about the patient’s smoking status. |
| hypertension | | present | hypertension_ present | The sentence indicates or implies that the patient has high blood pressure. |
|  |  | absent | hypertension_ absent | The sentence indicates or implies that the patient does not have high blood pressure. |
|  |  | unknown | hypertension_ unknown | The sentence does not contain any information about the patient’s hypertension status. |
| diabetes | | present | diabetes_present | The sentence indicates or implies that the patient has diabetes (any type). |
|  |  | absent | diabetes_absent | The sentence indicates or implies that the patient does not have diabetes. |
|  |  | unknown | diabetes_unknown | The sentence does not contain any information about the patient’s diabetes status. |
| heart failure | | present | heart_failure_ present | The sentence indicates or implies that the patient has suffered from heart failure (including single side heart failure). |
|  |  | absent | heart_failure_ absent | The sentence indicates or implies that the patient does not have suffered from heart failure. |
|  |  | unknown | heart_failure_ unknown | The sentence does not contain any information about the patient’s heart failure status. |
|  | | present | COPD_present | The sentence indicates or implies that the patient has COPD. |
| COPD |  | absent | COPD_absent | The sentence indicates or implies that the patient does not have COPD. |
|  |  | unknown | COPD_unknown | The sentence does not contain any information about the patient’s copd status. |
| family history of cancer |  | present | family_history_ present | The sentence indicates or implies that the patient has family history of cancer (any type). |
|  |  | absent | family_history_ absent | The sentence indicates or implies that the patient does not have family history of cancer. |
|  |  | unknown | family_history_ unknown | The sentence does not contain any information about the patient’s family history of cancer. |

##

## *Complementary criteria and examples*

1. Annotators should check 3 factors about a sentence before labeling it : the mention of the targeted medical condition; the subject concerned by the medical condition; the relationship between the medical condition and the subject. Only when all three factors in a sentence check the description of a medical status can it be annotated with a corresponding label.

• For example, to label a sentence with *smoking_former*, the annotator needs to confirm that smoking behavior or trace of tobacco poisoning is mentioned in the sentence, and its subject is the patient, and the sentence confirms the previous possession as well as the current non-possession of the condition by the patient.

• If the sentence does not mention the subject of a medical condition, by default it is considered as the patient.

**–** i.e. the sentence *Pas d’intoxication tabagique.* confirms that the patient does not smoke, and the sentence should be labeled as *smoking_absent*.

• If the information of medical condition in the sentence does not match the targeted one (or is missing), and/or the targeted subject


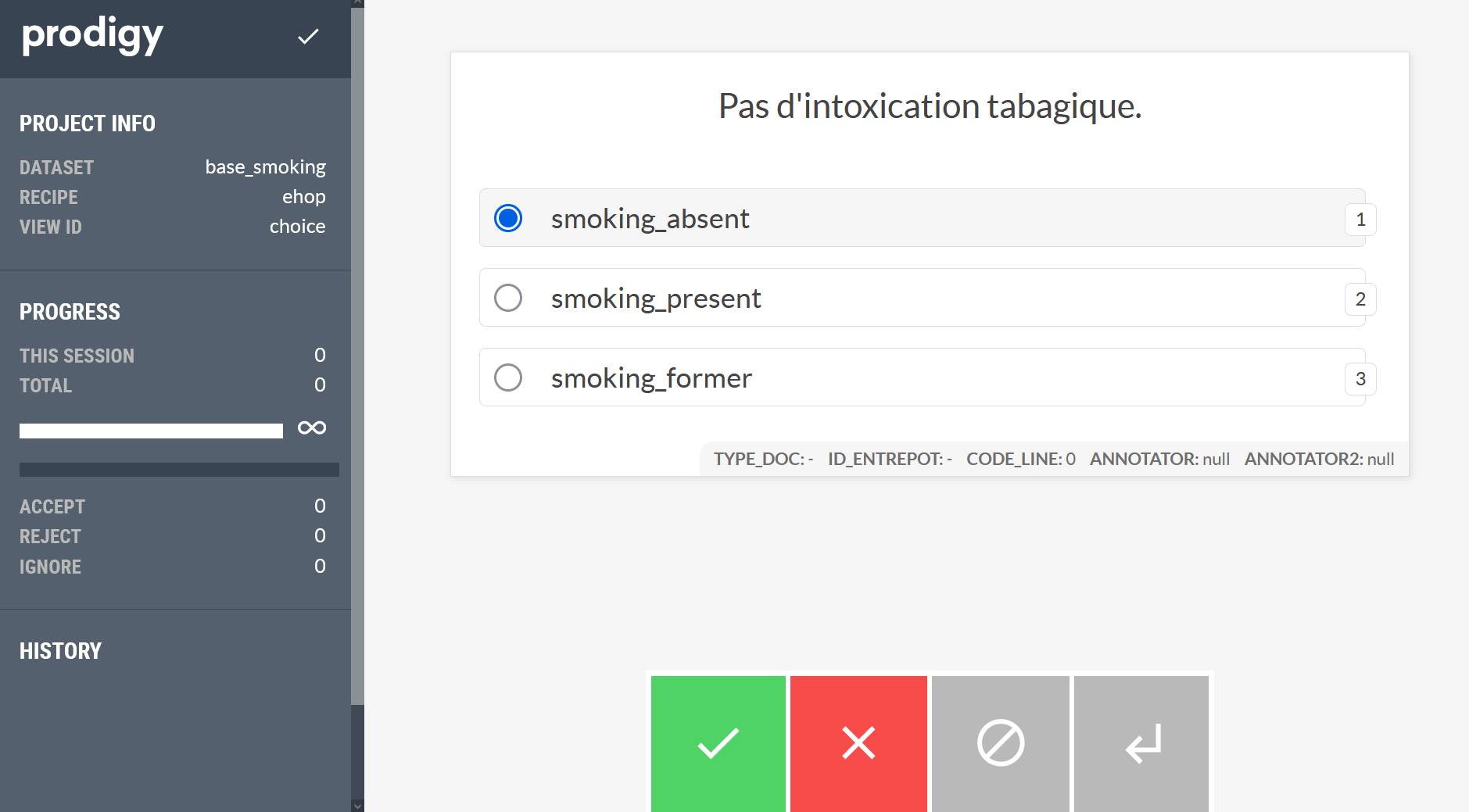


Figure S1: Example of annotating the smoking status of a sentence

(patient/family member) is not found, then the sentence should be classified as *unknown*.

**–** In order to shorten the time spent by annotators for choosing the labels, no choice for an unknown label is provided on the interface of annotation (as is shown in Figure A1). Instead by not choosing any of the other labels, the sentence is classified as *unknown* of the medical condition in question.

1. The extracted status should only concern the targeted medical condition instead of other medical conditions that are related to it but cannot be a direct proof of its presence/absence.
   - For example, mitral regurgitation, ostium secundum, capillary pressure–saturation are either non-definite causes or non-exclusive consequences of heart failure, therefore the presence of these medical conditions should not be considered as proof of a heart failure.
2. Certain medical status may be suggested in the sentence in an obscure way (medicine taken by the patient, the value of certain medical measurements). In that case, use your medical expertise to discover and confirm it. Make sure that these hints are direct and exclusive proof of the targeted medical condition.
   - A smoking cessation process, if not finished, implies that the patient is still an active smoker, the label should be *smoking_present*.
   - DPP-4 Inhibitors is a medicine exclusively used for treating diabetes. If the patient is taking it, it's proof that he/she has diabetes.
   - Normal blood pressure range is between 90/60 mmHg and 150/90 mmHg. If the sentence confirms that a patient has higher blood pressure than 150/90 mmHg, it can be labeled with *hypertension_present.*
3. All forms of medical conditions’ name should be taken into account when labeling, including abbreviation and conventional symbolic representation used by doctors.

• IC, ICD and ICG represent respectively ”insuffisance cardiaque” (heart failure), ”insuffisance cardiaque droite” (right-side heart failure) and ”insuffisance cardiaque gauche” (left-side heart failure), these abbreviations should be considered as a mention of the medical condition of *heart failure* when annotating a sentence.

• The expression ”atcd f k” is a conventional phrase used by doctors to represent ”antecedent familial de cancer” (family history of cancer).

1. If multiple status of a medical condition has been found in a sentence, the annotation should be based on the most recent one, except for *smoking_former*.

• For example, if in a sentence the patient had diabetes that are recently cured, then the sentence should be labeled with *diabetes_absent*; But if in a sentence, the patient used to smoke but recently severed, then the sentence should be labeled with *smoking_former* instead of *smoking_absent*.
