# Supplementary material for: A Sentence Classification–Based Medical Status Extraction Pipeline for Electronic Health Records: Institutional Case Study
Source: JMIR Med Inform. 2026 Mar 26;14:e77409. doi: 10.2196/77409 (PMC13044345; doi:10.2196/77409)
Supplement: Multimedia Appendix 3 [file medinform-v14-e77409-s003.docx]

**Multimedia Appendix 3**

**Complementary paired t-test evaluation of extractor performance across cross-validation folds**

This appendix presents complementary statistical analyses conducted to support the comparative evaluation of the three extractor types developed in this study: CamemBERT-based, rule-based, and LLM prompting based extractors. Paired t-tests were performed across the three folds of the cross-validation to assess whether observed performance differences (macro F-score) between these extractors for each medical condition were statistically significant (results presented in Table A3).

Table S1: Paired *t*-test results comparing the performance (macro F-score) of CamemBERT-based, rule-based and LLM (Mixtral) prompting extractors for all 6 medical conditions across 3-fold cross-validation (degree of freedom = 2). The t-statistic (t) and p-value (*P*) are reported in this table, complementing the means and standard deviations presented in Table 4. ^a^

|  | CamemBERT vs Rules | CamemBERT vs Mixtral | Rules vs Mixtral |
| --- | --- | --- | --- |
| smoking | t = 51.8  *P* < .001 | t = 18.3  *P =* .003 | t = -6.4  *P =* .02 |
| diabetes | t = 1.2  *P =* .4 | t = 5.9  *P =* .03 | t = 6.3  *P =* .02 |
| hypertension | t = 4.9  *P =* .04 | t = 13.9  *P =* .005 | t = 2.7  *P =* .11 |
| heart failure | t = 42.4  *P <* .001 | t = 11  *P =* .008 | t = -65.8  *P <* .001 |
| COPD | t = 0.87  *P =* .5 | t = 4.6  *P =* .04 | t = 6.7  *P =* .02 |
| family history of cancer | t = -1.3  *P =* .3 | t = -0.1  *P =* .9 | t = 0.9  *P =* .4 |

^a^The number of positive samples for family history of cancer is very small (n = 7); therefore, the reported p-values should be interpreted with caution due to insufficient sample size for robust statistical inference.

Across all comparisons, CamemBERT exhibits the strongest statistical performance. When evaluated against the rule-based system, it achieves extremely large and highly significant improvements for smoking (t = 51.8, *P* < .001), hypertension (t = 4.9, *P* = .04), and heart failure (t = 42.4, *P* < .001). For diabetes and COPD, advantages are not statistically significant (*P*=.4 and .5 respectively), indicating comparable performance. The rule-based system shows a numerical advantage over CamemBERT (t=–1.3) for family history of cancer, but the high p-value (*P* = .3) indicates that this superiority is not consistent or statistically reliable.

The comparison between CamemBERT and Mixtral is even more decisive. CamemBERT significantly outperforms Mixtral on smoking (t = 18.3, *P* = .003), diabetes (t = 5.9, *P* = .03), hypertension (t = 13.9, *P* = .005), heart failure (t = 11, *P* = .008), and COPD (t = 4.6, *P* = .04). Only family history of cancer shows no meaningful difference (t = –0.1, *P* = .9).

The comparison between Mixtral and the rule-based system yields mixed outcomes: Mixtral significantly outperforms the rules for smoking (t = –6.4, *P* = .02) and heart failure (t = –65.8, *P* < .001), whereas the rule-based system significantly outperforms Mixtral for diabetes (t = 6.3, *P* = .02) and COPD (t = 6.7, *P* = .02). For hypertension (t = 2.7, *P* = .11) and family history of cancer (t = 0.9, *P* = .4), the rule-based system shows slight numerical advantages, but the elevated p-values indicate that these differences are not consistent or statistically reliable.

Taken together, these paired t-test results show that CamemBERT is the most robust and reliable model, with statistically significant improvements over both Mixtral and the rule-based system across nearly all tasks. Mixtral and the rule-based system exhibit intermediate performance. Although the rule-based system shows slight numerical advantages over Mixtral on several statuses, the associated p-values indicate that these differences are not statistically reliable.
